# Genome skimming as an efficient tool for authenticating commercial products of the pharmaceutically important *Paris yunnanensis* (Melanthiaceae)

**DOI:** 10.1186/s12870-023-04365-x

**Published:** 2023-06-29

**Authors:** Nian Zhou, Lilei Tang, Pingxuan Xie, Ke Miao, Chengjin Yang, Haiyang Liu, Yunheng Ji

**Affiliations:** 1 CAS Key Laboratory for Plant Diversity and Biogeography of East Asia, Kunming Institute of Botany, Chinese Academy of Sciences, Kunming, China; 2 University of Chinese Academy of Sciences, Beijing, China; 3 School of Traditional Chinese Medicine, Guangdong Pharmaceutical University, Guangzhou, China; 4 Yunnan Baiyao Group, Chinese Medicinal Resources Co. LTD, Kunming, China; 5 State Key Laboratory of Phytochemistry and Plant Resources in West China, Kunming Institute of Botany, Chinese Academy of Sciences, Kunming, China; 6 Yunnan Key Laboratory for Integrative Conservation of Plant Species with Extremely Small Population, Kunming Institute of Botany, Chinese Academy of Sciences, Kunming, China

**Keywords:** Plastome, Rhizoma Paridis, Ribosomal DNA, Species identification

## Abstract

**Background:**

*Paris yunnanensis* (Melanthiaceae) is a traditional Chinese medicinal plant of significant pharmaceutical importance. Due to previous taxonomic confusion, a congeneric species, *Paris liiana*, has been mistaken for *P. yunnanensis* and cultivated on a large scale, leading to the mixing of commercial products (i.e., seedlings and processed rhizomes) of *P. yunnanensis* with those of *P. liiana.* This may have adverse effects on quality control in the standardization of *P. yunnanensis* productions. As the lack of PCR amplifiable genomic DNA within processed rhizomes is an intractable obstacle to the authentication of *P. yunnanensis* products using PCR-based diagnostic tools, this study aimed to develop a PCR-free method to authenticate commercial *P. yunnanensis* products, by applying genome skimming to generate complete plastomes and nrDNA arrays for use as the molecular tags.

**Results:**

Based on a dense intraspecies sampling of *P. liiana* and *P. yunnanensis*, the robustness of the proposed authentication systems was evaluated by phylogenetic inferences and experimental authentication of commercial seedling and processed rhizome samples. The results indicate that the genetic criteria of both complete plastomes and nrDNA arrays were consistent with the species boundaries to achieve accurate discrimination of *P. yunnanensis* and *P. liinna*. Owing to its desirable accuracy and sensitivity, genome skimming can serve as an effective and sensitive tool for monitoring and controlling the trade of *P. yunnanensis* products.

**Conclusion:**

This study provides a new way to solve the long-standing problem of the molecular authentication of processed plant products due to the lack of PCR amplifiable genomic DNA. The proposed authentication system will support quality control in the standardization of *P. yunnanensis* products in cultivation and drug production. This study also provides molecular evidence to clarify the long-standing taxonomic confusion regarding the species delimitation of *P. yunnanensis*, which will contribute to the rational exploration and conservation of the species.

**Supplementary Information:**

The online version contains supplementary material available at 10.1186/s12870-023-04365-x.

## Background

*Paris yunnanensis* (Melanthiaceae), a rhizomatous herbaceous perennial plant that is found in southwestern China and northern Myanmar, is a medicinal plant of great economic importance. The well-known traditional Chinese medicine, Rhizoma Paridis, is made from the rhizomes of *P. yunnanensis* and its congeneric species *Paris chinensis* [1] (hereafter referred to as ‘medicinal *Paris*’). For more than 2,000 years, Rhizoma Paridis has been used to treat more than 30 diseases, such as asthma, bleeding, fever, flu, infection, injuries, insect bites, mumps, rheumatic pain syndrome, pruritus, snakebites, swelling, tonsillitis, and ulcers [2, 3]. Recently, modern pharmacological investigations have successively discovered its anti-inflammatory, analgesic, antipyretic, antitumor, immunostimulatory, antimicrobial, and other therapeutic properties [2, 4]. On this basis, more than 90 commercial drugs and health products using Rhizoma Paridis as a raw material have been developed in China [2]. The annual revenue generated by these pharmaceutical products was estimated to be approximately 10 billion CNY (ca. 1.6 billion USD) [5].

In contrast to the substantial demand and consumption of raw materials for drug production (approximately 3 × 10^6^ kg per year), the natural propagation and growth of medicinal *Paris* and congeneric species are very slow [2, 3, 6, 7]. Species with slow growth rates are generally more prone to overexploitation [8, 9], and the large-scale harvesting of rhizomes from the wild in recent decades has inevitably led to sharp decreases in the natural populations of both *P. chinensis* and *P. yunnanensis* [2–6, 10]. With the continuous depletion of natural resources, the pharmaceutical industry that uses Rhizoma Paridis as a raw material has encountered a serious supply crisis [3, 6, 10]. Under this circumstance, the commercial cultivation of medicinal *Paris* has rapidly expanded in China since the early 2000s, with *P. yunnanensis* being cultivated on the largest scale to produce raw material for drug production [2, 3].

Notably, *P. yunnanensis* is highly variable in terms of petal color, folia size and shape, and pedicel length; therefore, the taxonomic delimitation of the species has frequently changed [2, 11–15]. Recently, a total of 4 nominal taxa (i.e., *Paris birmanica*, *Paris daliensis*, *Paris polyphylla* var. *emeiensis*, and *Paris polyphylla* var. *nana*) have been synonymized to *P. yunnanensis* based on morphological analyses [2, 14]. Inferred from morphological and molecular evidence, a previous study revealed that *P. yunnanensis* is a collective species consisting of two morphologically distinct and genetically disparate lineages that have little overlap between their distribution ranges; as a result, a new species, *Paris liiana*, was discovered and described [15]. Due to the previous ambiguity in species delineation, *P. liiana* has been mistaken for and cultivated as *P. yunnanensis* on a large scale (Fig. S1). This inevitably leads to the commercial products of *P. yunnanensis* (i.e., seedlings and processed rhizomes) being mixed with those of *P. liiana.* In view of the differences in the rhizome chemical compositions and contents between the two species [2, 4, 16], the mixing with *P. liiana* may have adverse impacts on quality control in either the standardized cultivation of *P. yunnanensis* or drug production. Given the great importance of *P. yunnanensis* to the pharmaceutical industry, there is an urgent need for an authentication method that can accurately and sensitively detect contaminating *P. liiana* among the commercial products of *P. yunnanensis*.

**Fig. 1:**
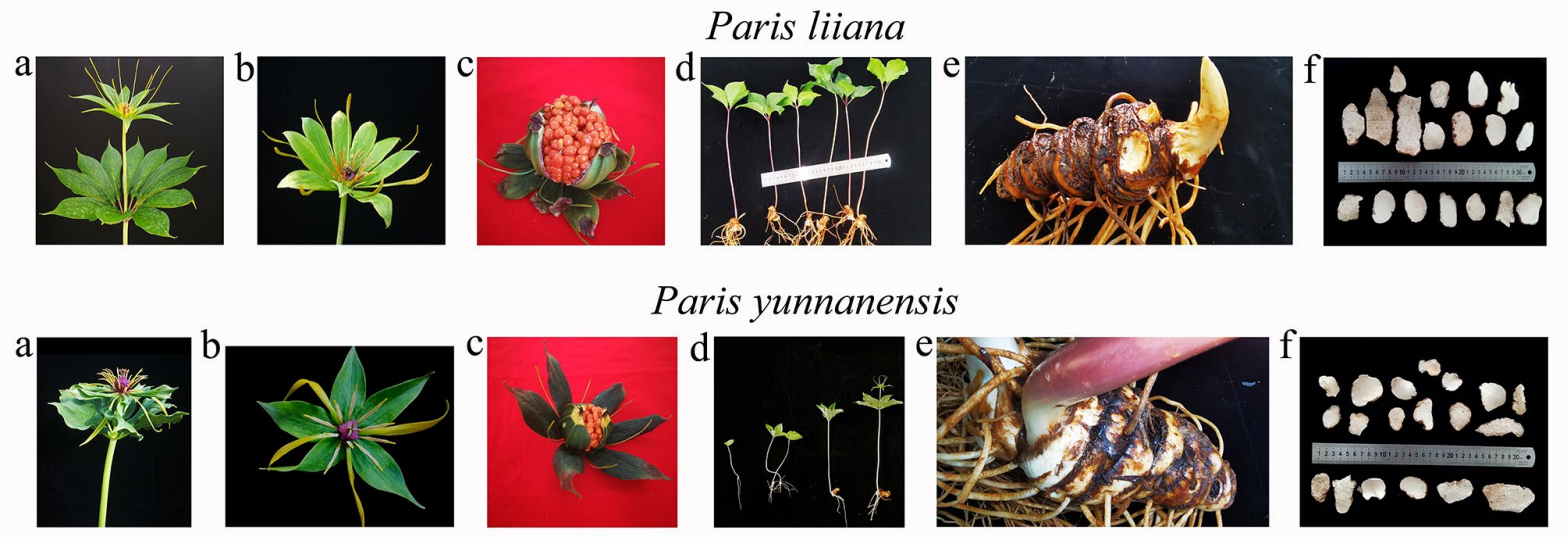
Morphologies of arial shoot **(a)**, flower **(b)**, fruit **(c)**, seedling **(d)**, rhizome **(e)**, and processed rhizomes **(f)** of *Paris yunnanensis* and *Paris liiana*

Although *P. liiana* and *P. yunnanensis* are easily distinguished based on the differences in flower and fruit morphologies [2, 15], their seedling and processed rhizomes are morphologically indistinguishable (Fig. 1), making the morphological identification of such commercial products difficult. Since the beginning of this century, interspecific DNA sequence variations have been commonly used for species discrimination [17–23]. To date, many PCR-based diagnostic tools, such as standard (or lineage-specific) DNA barcodes [10, 24–27], RAPD fingerprints [28], and sequence-characterized amplified region (SCAR) markers [29–34], have been developed for authenticating the commercial products of medicinal plants. Given that genomic DNA of some commercial plant products have been highly degraded during processing, the lack of PCR amplifiable genomic DNA within the processed botanic materials becomes an intractable obstacle to the application of these diagnostic tools that exclusively rely on PCR to recovered target DNA regions for species identification [35–39].

The introduction and development of high-throughput sequencing technologies has made the identification of plant species no longer dependent on PCR [35, 40, 41]. Genome skimming, a PCR-free approach that recovers complete plastid genomes (plastomes) and nuclear tandemly repeated ribosomal DNA (nrDNA) arrays (18 S rDNA–ITS1–5.8 S rDNA–ITS2–26 S rDNA) as the molecular tags from shallow-depth genome sequencing data, has been recommended as an alternative method for plant species identification [21, 35, 42, 43]. Compared with single or multiple sequence regions generated by Sanger sequencing, either complete plastomes or nrDNA arrays possess far more variable loci; therefore, the application of both datasets in plant species discrimination is considered to have great potential for improving resolution [21, 23, 35, 40–43]. Consistent with this theoretical expectation, empirical studies have shown that although the application of genome skimming is not yet robust enough to distinguish all species, especially in evolutionarily young and complex plant taxa, it can substantially improve the efficacy of species discrimination in most of the studied taxa [38, 41, 44–52]. Better yet, previous studies have shown that even using trace and highly degraded genomic DNA extracted from processed plant materials to build shotgun libraries, complete plastomes and nrDNA arrays can be recovered by application of high-throughput sequencing technologies [35, 37, 38], and the use of genome skimming has shown desirable performance in accurate and sensitive identification of species in mixed samples [53, 54].

In view of the favorable advantages of high-throughput sequencing technologies in terms of species identification, this study aimed to develop a PCR-free method for effective authenticating commercial seedlings and processed rhizomes of *P. yunnanensis* by employing genome skimming to generate complete plastomes and nrDNA arrays for use as the molecular tags. To achieve this goal, individual plants representing the phenotypic diversity of *P. liiana* and *P. yunnanensis* were densely sampled and subjected to genome skimming to generate complete plastome and nrDNA array for each sample. On this basis, a reference dataset for the discrimination of *P. liiana* and *P. yunnanensis* was built, and the efficacy of genome skimming in identifying the source species of commercial seedling and processed rhizome samples was empirically verified.

## Results

### Illumina sequencing and recovery of complete plastomes and nrDNA arrays

Illumina sequencing yielded approximately 12.10‒40.61 million paired-end (2 × 150 bp) clean reads per sample, and based on these reads, the reference-guild assembly recovered the complete plastome and entire nrDNA arrays for each sample. These newly generated plastomes (varied from 157,674 to 158,384 bp in length, with average sequencing coverage ranging from 286 to 6,016 times; Table S4) possessed an identical quadripartite structure that consisted of a large single-copy (LSC) and a small single-copy (SSC) separated by two copies of inverted repeat (IR) regions (Table S4 & Table S5). The assembly of the nrDNA arrays completely recovered 18 S rDNA, ITS1, 5.8 S rDNA, ITS2, and 26 rDNA regions for each sample, with the sequence length varying from 5,836 to 5,852 bp and the average sequencing coverage ranging from 375 to 1,487 times (Table S4 & Table S5). The plastomes and nrDNA arrays generated in this study were deposited in the NCBI GenBank database, and their accession numbers are shown in Table S1.

### Complete plastomes and nrDNA arrays for discriminating between Paris ***liiana*** and ***Paris yunnanensis***

**Fig. 2:**
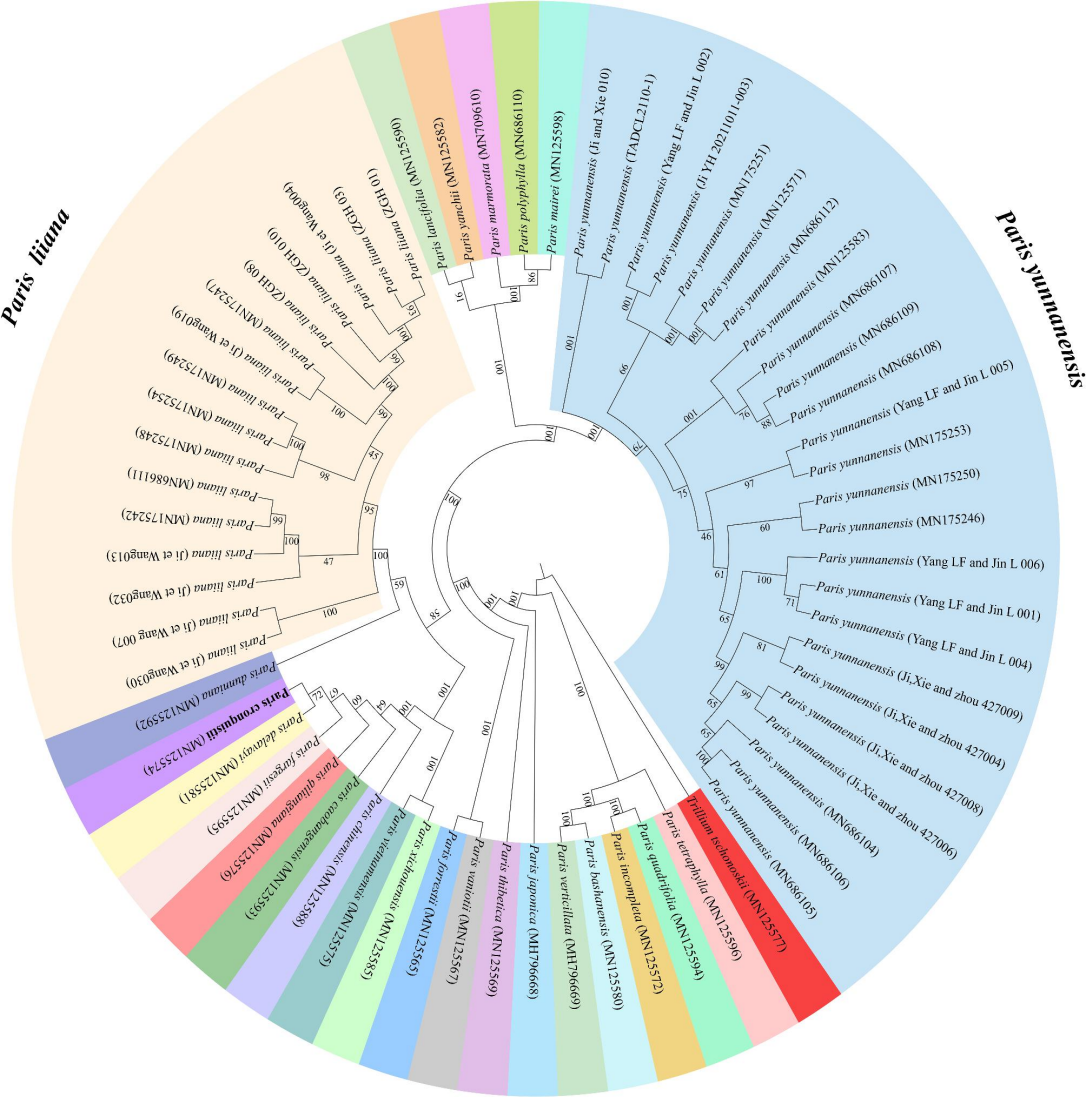
Cladogram of the maximum-likelihood (ML) phylogeny resulted from analyzing the complete plastomes of *Paris* species. The numbers at nodes represents ML bootstrap (BS) percentages

**Fig. 3:**
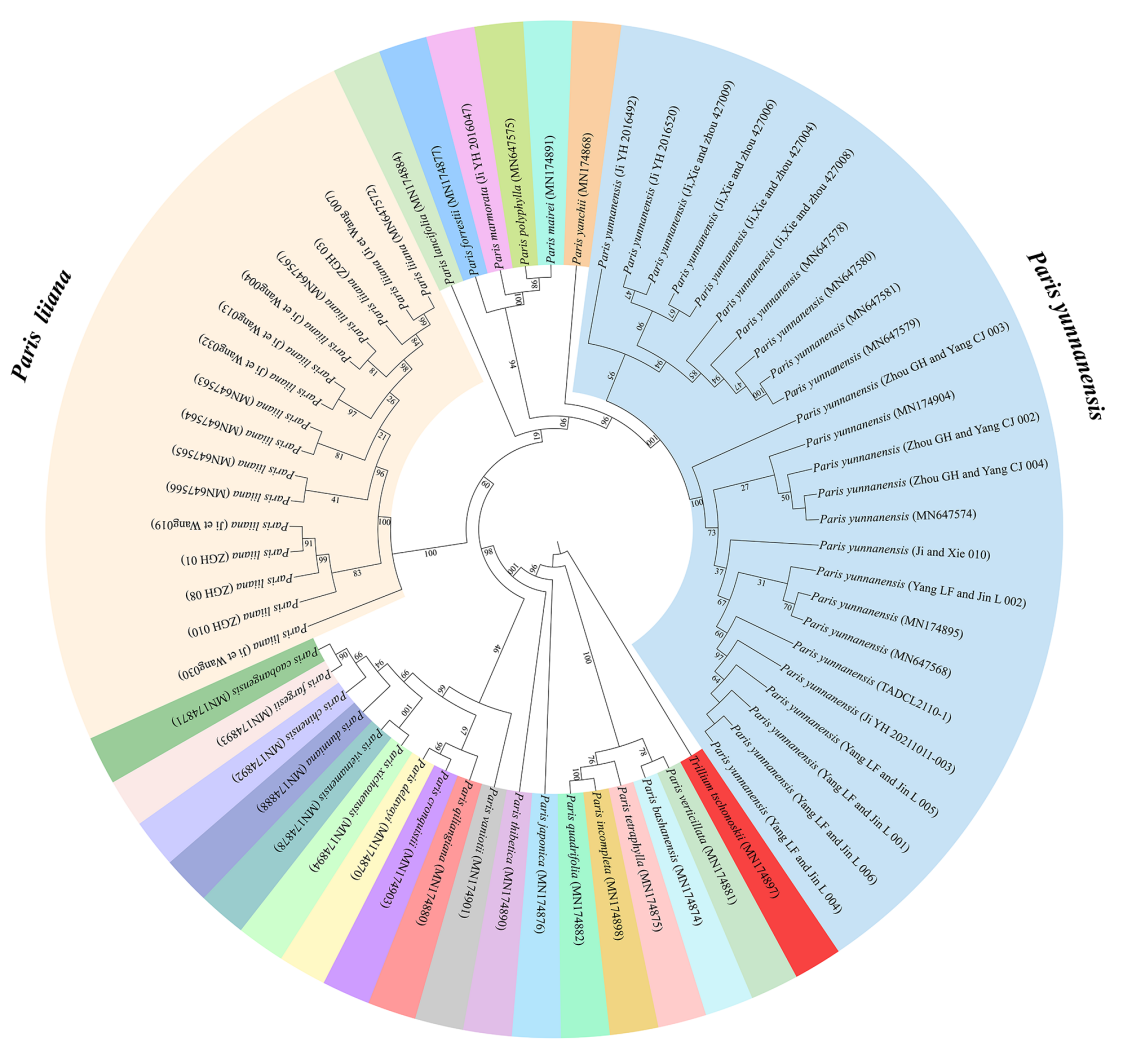
Cladogram of the maximum-likelihood (ML) phylogeny resulted from analyzing the nuclear ribosome DNA (nrDNA) arrays of *Paris* species. The numbers at nodes represents ML bootstrap (BS) percentages

Based on intensive intraspecific sampling, the efficacy of the complete platomes and nrDNA arrays in discriminating between *P. liiana* and *P. yunnanensis* was evaluated by examining whether and to what extent each dataset matches the species boundaries. Briefly, ML analysis of complete plastomes (Fig. 2) resolved both species as fully supported monophyletic entities (BS = 100%), and the two species were phylogenetically distinct from each other in the tree topology. Similar to previous studies [49, 55], ML analysis of the nrDNA arrays generated tree topologies that were completely different from the plastome phylogeny (Fig. 3). Even so, both *P. liiana* and *P. yunnanensis* were identically resolved as monophyletic entities with full branch support (BS = 100%) by the nrDNA phylogeny. Additionally, neither plastome nor nrDNA phylogeny resolved the four synonymized taxa, i.e., *P. birmanica*, *P. daliensis*, *P. polyphylla* var. *emeiensis*, and *P. polyphylla* var. *nana*, as monophyletic entities but embedded them within accessions of “Typical” *P. yunnanensis* in the ML trees (Fig. 2 & Fig. 3). The well-supported species-level monophyly of *P. liiana* and *P. yunnanensis* showed that the genetic criteria of either the plastome or nrDNA dataset are consistent with the species boundaries of these two species.

### Experimental authentication of commercial seedling and processed rhizome samples

**Fig. 4:**
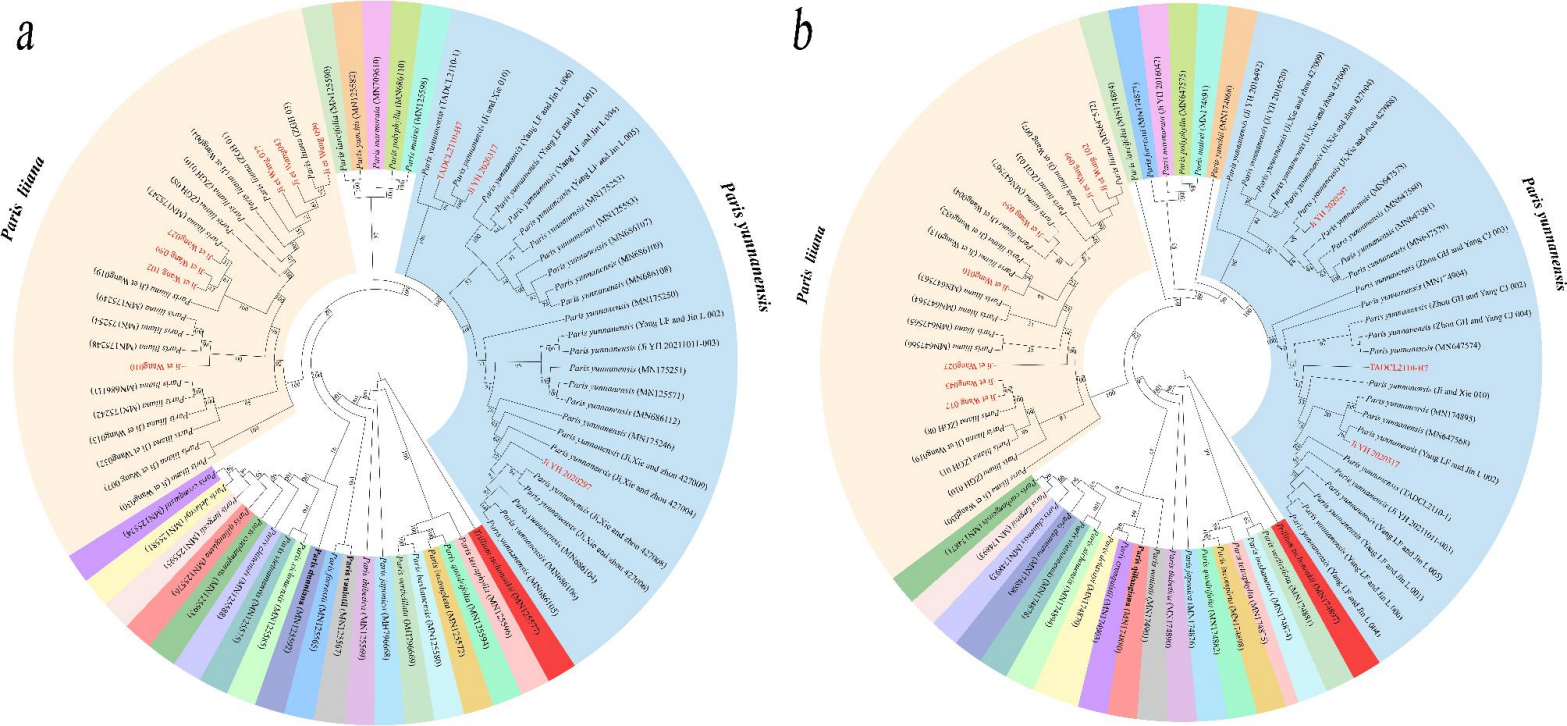
Species assignment of the commercial seedlings marketed as *Paris yunnanensis* (highlighted in red) via maximum-likelihood (ML) analyses of complete plastomes **(a)** and nuclear ribosome DNA (nrDNA) arrays **(b)**. The numbers at nodes represents ML bootstrap (BS) percentages

**Fig. 5:**
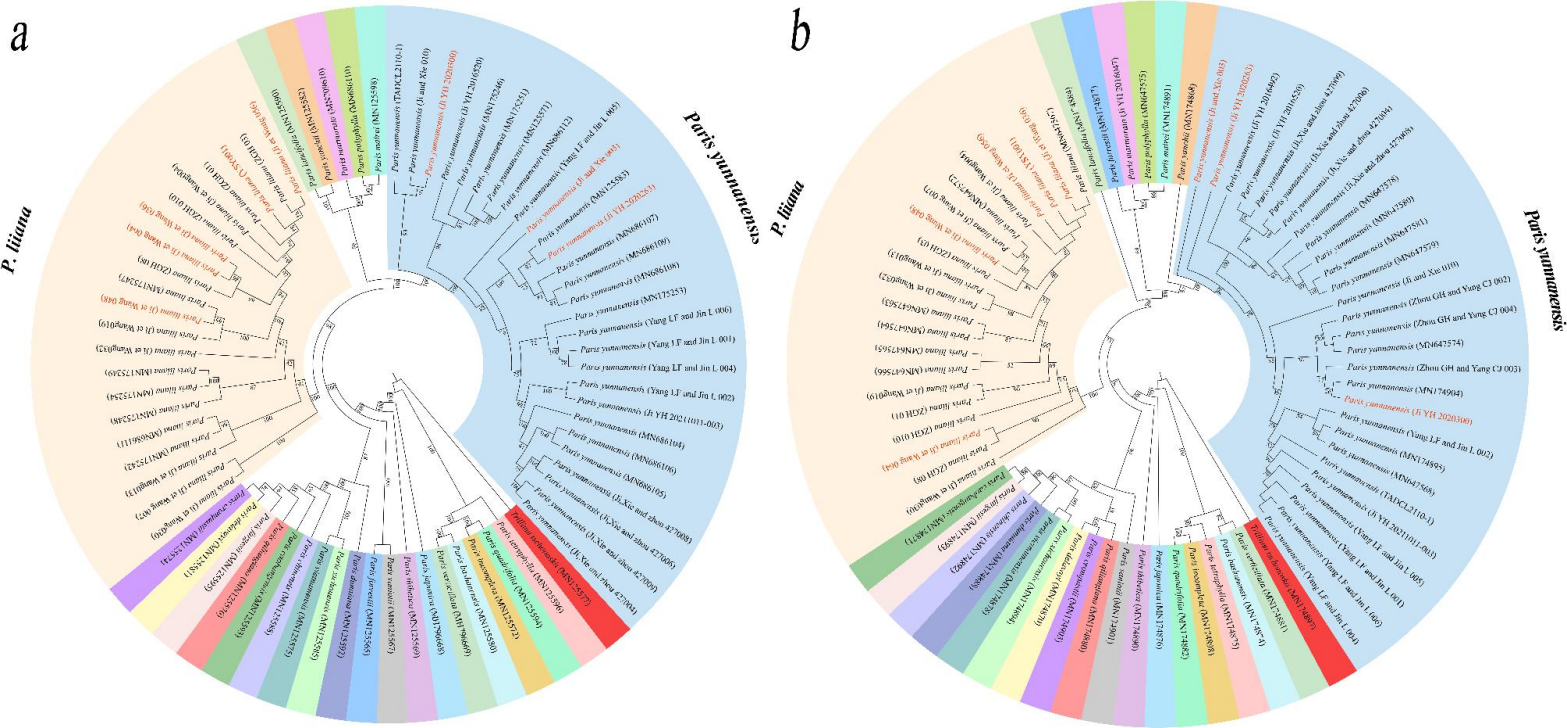
Species assignment of the processed rhizomes marketed as *Paris yunnanensis* (highlighted in red) via maximum-likelihood (ML) analyses of complete plastomes **(a)** and nuclear ribosome DNA (nrDNA) arrays **(b)**. The numbers at nodes represents ML bootstrap (BS) percentages

Based on the reference plastome and nrDNA datasets, ML phylogeny was determined in order to identify the species to which the commercial seedling and rhizome samples belong. For the commercial seedlings marketed as *P. yunnanensis*, the ML phylogeny assigned these commercial products to two distinct species. Among them, only three seedlings (Fig. 4) and three process rhizomes (Fig. 5) were clustered with *P. yunnanensis* (BS = 100%), while seven seedling (Fig. 4) and five rhizome (Fig. 5) samples were clustered with *P. liiana* (BS = 100%). As the aggregation of query sequences and reference sequences was well supported, the species assignment scheme proposed by phylogenetic analysis can be considered credible [56].

## Discussion

### Genome skimming for authenticating the commercial products of ***Paris yunnanensis***

The primary goal of the standardized cultivation of medicinal plants is to produce high-quality raw materials that meet therapeutic requirements, which means that quality control should be prioritized in the production of medicinal plants [57, 58]. Due to previous taxonomic ambiguity, commercial seedlings and processed rhizomes of *P. yunnanensis* are likely mixed with those of *P. liiana*, which has adverse impacts on quality control in either the standardized cultivation of *P. yunnanensis* or drug production [58]. Therefore, the detection of *P. liiana* among the commercial products of *P. yunnanensis* is crucial for the standardization of *P. yunnanensis* productions. Given the great importance of *P. yunnanensis* to the pharmaceutical industry, an accurate and sensitive authentication method is urgently needed to distinguish the commercial seedlings and processed rhizomes of *P. yunnanensis* from those of *P. liiana*. In view of the intractable obstacle of PCR-based diagnostic tools in authenticating such commercial plant products (i.e., the processed rhizomes of *Paris* species) whose genomic DNA have been highly degraded during processing [35–39], this study aimed to develop a PCR-free method for accurate and effective authentication of the commercial seedlings and processed rhizomes of *P. yunnanensis*, using genome skimming to generate complete plastomes and nrDNA arrays for use as the molecular tags.

The availability of a high-quality reference dataset is an indispensable prerequisite for the application of genome skimming for species discrimination [35, 59–62]. Accordingly, the creation of robust reference complete plastome and nrDNA datasets is the essential first step in the practical application of genome skimming for the authentication of *P. yunnanenis* and its commercial products. In this study, the complete plastomes and nrDNA arrays of 25 *P. yunnanensis* and 16 *P. liinna* individuals, representing the intraspecific phenotypic diversity of both species, were sampled to construct the reference dataset. These sequences were obtained from correctly identified specimens, and the sequences constituted comprehensive and high-quality complete reference plastome and nrDNA datasets. Additionally, phylogenetic analysis of both datasets resolved *P. yunnanensis* and *P. liinna* as monophyletic entities with high statistical supports, indicating that the genetic criteria of complete plastomes and nrDNA arrays were consistent with the species boundaries of *P. yunnanensis* and *P. liinna*. This suggests that accurate discrimination of *P. yunnanensis* and *P. liinna* can be achieved using either complete plastomes or nrDNA arrays as the molecular tags.

To validate the efficacy of the proposed authentication system, this study experimentally applied genome skimming to identify the source species of commercial seedling and processed rhizome samples. Although the processed rhizomes of *Paris* species represent botanic materials with highly degraded genomic DNA, the complete plastomes and nrDNA arrays of all samples were recovered from Illumina reads. The desirable success rate of recovering complete plastomes and nrDNA arrays suggests that they are ideal molecular tags for authentication the processed rhizomes of *Paris yunnanensis*. Based on the high-quality reference plastome and nrDNA datasets, ML phylogeny unambiguously detected *P. liiana* among commercial seedling and processed rhizomes marketed as *P. yunnanensis*, validating the desired accuracy and practicality of the proposed authentication system.

To date, numerous PCR-based diagnostic tools have been developed for authenticating the commercial products of medicinal plants [10, 24–34]. Although these methods exhibit the merits of time and cost effectiveness, the success of their authentication depends largely on the DNA quality and integrity of examined samples. This should limit their practical application in the authentication of some processed plant products, where genomic DNA have been highly degraded during processing [35–39].

The current study represents the first case to develop a PCR-free method to authenticate commercial products of *P. yunnanensis*, a pharmaceutically important plant species. Although considerable bioinformatic skills are needed to recover complete plastomes and nrDNA arrays from low coverage genome sequencing data and to perform phylogenetic analyses, the method developed in this study provides a good solution for authentication of processed plant products that lack PCR amplifiable genomic DNA. Recently, the advances in high-throughput sequencing technology have largely decreased the cost of low coverage genome sequencing, and the development of new approaches have made it easier to recover complete plastomes and nrDNA arrays from shotgun sequencing data [63–66]. Accordingly, the authentication system developed in this study can serve as an effective and sensitive tool for monitoring and controlling the trade of seedlings and processed rhizomes, which will contribute to quality control in the standardized cultivation of *P. yunnanensis* and drug production.

### Implications for conservation and exploitation of ***Paris yunnanensis***

**Fig. 6:**
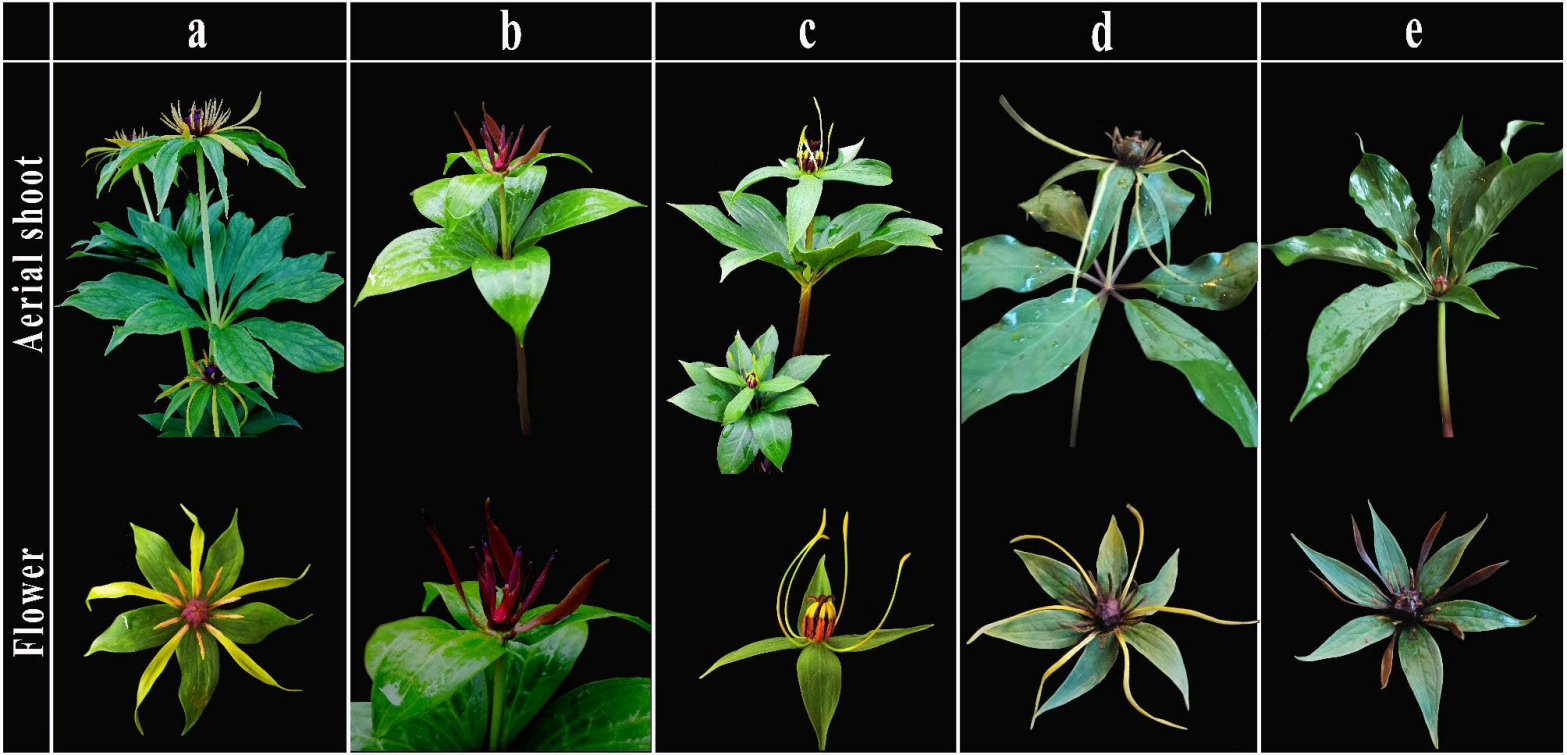
Morphology of “typical” *Paris yunnanensis ***(a)** and synonymized taxa: *Paris birmanica ***(b)**, *Paris daliensis ***(c)**, *Paris polyphylla* var. *nana ***(d)**, and *Paris polyphylla* var. *emeiensis ***(e)**

This study provides further evidence supporting the taxonomic revision that considered *P. birmanica*, *P. daliensis*, *P. polyphylla* var. *emeiensis*, and *P. polyphylla* var. *nana* to be synonyms for *P. yunnanensis* [2, 14]. Specifically, neither plastome nor nrDNA phylogeny resolved the four synonymized taxa, i.e., *P. birmanica*, *P. daliensis*, *P. polyphylla* var. *emeiensis*, and *P. polyphylla* var. *nana*, as monophyletic entities but embedded them within accessions of “Typical” *P. yunnanensis* in the phylogenetic trees, suggesting that these taxa have not genetically diverged from “Typical” *P. yunnanensis*. Accordingly, either recognizing *P. birmanica* and *P. daliensis* as segregated species [12, 67] or treating *P. polyphylla* var. *emeiensis* and *P. polyphylla* var. *nana* as conspecific varieties of *P. polyphylla* [68, 69] is likely a taxonomic error. This phylogenetic inference is in good agreement with morphological evidence: except for the purple petals of *P. birmanica* [12, 68, 70], the thickened free portion of the connectivum in *P. daliensis* [67], a nearly absent pedicel in *P. polyphylla* var. *emeiensis* [69], and an extremely shortened (less than 5 cm) pedicel in *P. polyphylla* var. *nana* [68], the four taxa are highly similar to “typical” *P. yunnanensis* in terms of their leaf and flower morphologies (Fig. 6). This suggests that the diagnostic characteristics that were used to define the four taxa may represent intraspecific morphological variations or phenotypic plasticity [2, 14], justifying to synonymize *P. birmanica*, *P. daliensis*, *P. polyphylla* var. *emeiensis*, and *P. polyphylla* var. *nana* with *P. yunnanensis* [2, 14].

On the other hand, *P. yunnanensis* is a medicinal plant whose conservation has attracted substantial concerns, as overexploitation of the species for medicinal purposes has resulted in the rapid depletion of its natural populations [2, 3]. Given that accurate delineation of species boundaries is the essential first step to properly address issues regarding species conservation and exploitation [71–73], this study clarifies the long-standing taxonomic confusion and provides robust phylogenetic evidence to delineate a clear-cut species boundary of *P. yunnanensis*, thus has great potential to improve strategies for the conservation and exploitation of the economically important species. Specifically, this study determines that previous taxonomic confusion has resulted in the proliferation of as many as four synonyms in *P. yunnanensis*. Among them, *P. daliensis* is categorized as critically endangered [74], *P. polyphylla* var. *emeiensis* and *P. polyphylla* var. *nana* are classified as endangered [69, 74], and *P. birmanica* is classified as vulnerable [75]. This suggests that previous taxonomic errors may have resulted in the inappropriate allocation of limited conservation resources to these taxa that are not at high risk of extinction. Additionally, previous taxonomic errors in species delimitation must have led to the omission of these synonymized taxa from the introduction and cultivation of *P. yunnanensis* and its germplasm conservation. Therefore, individual plants and populations of both “typical” *P. yunnanensis* and these synonymized taxa should be simultaneously collected to preserve the germplasm resources, from which elite germplasm can be excavated for breeding needs.

The collection and preservation of propagules is the preferred strategy for the conservation of plant germplasm resources [76, 77]. For the propagules (i.e., rhizomes, seeds, and seedlings) of *P. yunnanensis*, traditional morphology-based species identification is a fairly difficult task due to the absence of diagnostic morphologies in these organs. Nevertheless, based on the reference datasets of complete plastome and nrDNA arrays, determining whether propagules that will be collected and preserved belong to *P. yunnanesis* can be easy: genome skimming can be applied to recover their complete plastomes or nrDNA arrays, and then, these sequences can be queried against the corresponding reference dataset.

## Conclusions

For the first time, this study developed a PCR-free authentication system for detecting whether the commercial seedlings and processed rhizomes of *Paris yunnanensis*is are adulterated with the congeneric species, *Paris liiana*, by applying genome skimming to generate complete plastomes and nrDNA arrays for use as the molecular tags. Although the authentication system relies heavily on bioinformatic skills and is not time- and cost-efficient, this study provides a new way to solve the long-standing problem of the molecular authentication of processed plant products due to their lack of PCR amplifiable genomic DNA. Owing to its desirable accuracy and sensitivity, the authentication system will be conductive to the standardization of *P. yunnanensis*is products to guarantee their quality and effectiveness.

## Methods

### Plant sampling, Illumina sequencing, and recovery of complete plastomes and nrDNA arrays

In this study, the complete plastomes and nrDNA arrays of 25 *P. yunnanensis* and 16 *P. liinna* individuals were sampled to establish the reference dataset for the use of genome skimming to distinguish between *P. liinna* and *P. yunnanensis*. Among them, 12 and 10 individual plants, whose morphological characteristics represent the phenotypic diversity of *P. yunnanensis* and *P. liinna*, respectively, were sequenced for the first time in this study. The plant materials sampled in this study was identified by Dr. Yunheng Ji. The voucher specimens were deposited at herbarium of Kunming Institute of Botany (Chinese Academy of Sciences), and the original sources of the plant samples and voucher information are presented in Table S1. The remaining complete plastomes and nrDNA arrays were obtained from the NCBI GenBank database (Table S2). Notably, the intraspecific sampling of *P. yunnanensis* included not only individual plants with the “typical” morphological characteristics of the species but also individuals that morphologically represented the four synonymized taxa (Table S1), i.e., *Paris birmanica* (3 individuals), *Paris daliensis* (3 individuals), *Paris polyphylla* var. *emeiensis* (2 individuals), and *Paris polyphylla* var. *nana* (2 individuals), which were reduced to *P. yunnanensis* by Liang and Soukup [14] and Ji [2]. In addition, to experimentally validate the efficacy of genome skimming for the detection of *P. liiana* among the commercial seedlings and rhizomes of *P. yunnanensis*, a total of 10 commercial seedlings and eight processed rhizome samples marketed as *P. yunnanensis* (voucher information is presented in Table S3) were sampled for shotgun sequencing.

The genomic DNA of these samples was extracted with the CTAB method [78]. Illumina sequencing libraries with an average insert size of approximately 400 bp were built using a TruSeq DNA PCR-free Prep Kit (Illumina Inc., San Diego, CA, USA) according to the manufacturer’s protocol and then subjected to paired-end sequencing on an Illumina Novaseq 6000 platform to generate approximately two Gbp of raw reads for each sample. The software Trimmomatic v0.40 [79] was used to remove adaptors and to filter low-quality reads with default parameters. A reference-guided method was used for the assembly of complete plastomes and nrDNA arrays for each sample; the published complete plastome (GenBank accession: MN175247) and nrDNA sequence (GenBank accession: MN647572) of *P. liiana* were used as the reference, and the GetOrganelle v1.7.5.0 pipeline [66] was use to recover complete plastomes and nrDNA arrays from the trimmed Illumina reads, with default parameters and preset options. The obtained plastomes were annotated with the online program Geseq v2.03 [80] and further validated by performing a BLAST search against the NCBI protein database with Geneious v10.2.3 [81]. For the nrDNA arrays, the 28 S, 18 S, and 5.8 S ribosomal RNA genes and their boundaries with the intergenic transcribed spacer (ITS1 and ITS2) regions were annotated and defined by comparison with the reference sequence in Geneious v10.2.3 [81].

### Data analysis

According to phylogenetic tree topologies, the robustness of the complete plastomes and nrDNA arrays for the discrimination of *P. liiana* and *P. yunnanensis* was assessed by examining the monophyly of each species. In addition to the reference sequences of *P. liiana* and *P. yunnanensis*, 24 complete plastomes and nrDNA arrays that represent the remaining congeneric species were included in phylogenetic analysis (Table S2), with *Trillium tschonoskii* as the outgroup, according to the findings of previous studies [55, 82–84]. The complete plastomes and nrDNA arrays were independently aligned with MAFFT v7.402 [85] using the default parameters. For each dataset, phylogenetic relationships were reconstructed using the maximum likelihood (ML) method. The ML analyses were conducted with the online software IQ-TREE v2.2.0 [63, 64], and 1,000 ultrafast bootstrap (BS) replicates were performed to generate the percentage of BS support for each node.

To investigate whether genome skimming enables the accurate and sensitive detection of *P. liiana* among the commercial seedlings and rhizomes of *P. yunnanensis*, complete plastomes and nrDNA arrays from 10 commercial seedling and eight processed rhizome samples marketed as *P. yunnanensis* were incorporated into the plastome and nr DNA datasets to reconstruct the ML phylogeny. For each dataset, ML analyses were conducted using the previously described protocol. According to tree topology, the phylogenetic placement of each sample was investigated to determine whether the ML phylogeny accurately assigned these samples to their source species. In general, if the query sequence was clustered with the reference sequences from a certain taxon with high branch support, credible species assignment was considered to have been achieved [56].

## Electronic supplementary material

Below is the link to the electronic supplementary material.

Supplementary Material 1

Supplementary Material 2

Supplementary Material 3

Supplementary Material 4

Supplementary Material 5

Supplementary Material 6

## Data Availability

The complete plastomes and nrDNA arrays generated in this study are available at the NCBI GenBank database, with the following accession numbers: OP021412, OP021413, OP021414, OP021415, OP021474, OP021475, OP021476, OP021477, OP021478, OP021493, OP021494, OP021495, OP021496, OP021793, OP021797, OP021802, OP021804, OP021806, OP021817, OP021820, OP021823, OP021830, OP021831, OP021835, OP021843, OP021845, OP806312, OP806313, OP806314, OP806315, OP806316, OP806317, OP837990, OP837991, OP837992, OP837993, OP837994, and OP837995.

## Abbreviations

bp: Base pair
BP: Bootstrap percentage
BS: Bootstrap
CTAB: Cetyl trimethylammonium bromide
DNA: Deoxyribonucleic acid
Gb: Giga base pairs
IR: Inverted repeat
ITS: internal transcribed spacer of nuclear ribosomal DNA
LSC: Large single-copy
MCMC: Markov Chain Monte Carlo
ML: Maximum Likelihood
PP: Posterior probability
rRNA: Ribosomal RNA
SSC: Small single copy
tRNA: Transfer RNA
