# Supplementary material for: Genome skimming as an efficient tool for authenticating commercial products of the pharmaceutically important Paris yunnanensis (Melanthiaceae)
Source: BMC Plant Biol. 2023 Jun 29;23:344. doi: 10.1186/s12870-023-04365-x (PMC10308783; doi:10.1186/s12870-023-04365-x)
Supplement: Supplementary file 1 — Supplementary Material 1 [file 12870_2023_4365_MOESM1_ESM.docx]

**Table S2.** Publicly available plastomes and nrDNA arrays obtained from NCBI GenBank

| Samples | Genbank accessions | |
| --- | --- | --- |
|  | Plastome | Ribosomal DNA |
| *Paris bashanensis* | MN125580 | MN174874 |
| *Paris caobangensis* | MN125593 | MN174871 |
| *Paris chinensis* | MN125588 | MN174892 |
| *Paris cronquistii* | MN125574 | MN174903 |
| *Paris delavayi* | MN125581 | MN174870 |
| *Paris dunniana* | MN125592 | MN174888 |
| *Paris fargesii* | MN125595 | MN174893 |
| *Paris forrestii* | MN125565 | MN174877 |
| *Paris incompleta* | MN125572 | MN174898 |
| *Paris japonica* | MH796668 | MN174876 |
| *Paris lancifolia* | MN125590 | MN174884 |
| *Paris liiana* | MN175242 | MN647567 |
| *Paris liiana* | MN175247 | MN647572 |
| *Paris liiana* | MN175248 | MN647564 |
| *Paris liiana* | MN175249 | MN647563 |
| *Paris liiana* | MN175254 | MN647565 |
| *Paris liiana* | MN686111 | MN647566 |
| *Paris mairei* | MN125598 | MN174891 |
| *Paris polyphylla* | MN686110 | MN647575 |
| *Paris qiliangiana* | MN125576 | MN174880 |
| *Paris quadrifolia* | MN125594 | MN174882 |
| *Paris tetraphylla* | MN125596 | MN174875 |
| *Paris thibetica* | MN125569 | MN174890 |
| *Paris vaniotii* | MN125567 | MN174901 |
| *Paris verticillata* | MH796669 | MN174881 |
| *Paris vietnamensis* | MN125575 | MN174878 |
| *Paris xichouensis* | MN125585 | MN174894 |
| *Paris yanchii* | MN125582 | MN174868 |
| *Paris yunnanensis* | MN125571 | MN174895 |
| *Paris yunnanensis* | MN125583 | MN174904 |
| *Paris yunnanensis* | MN175246 | MN647578 |
| *Paris yunnanensis* | MN175253 | MN647574 |
| *Paris yunnanensis* | MN686104 | MN647579 |
| *Paris yunnanensis* | MN686105 | MN647580 |
| *Paris yunnanensis* | MN686106 | MN647581 |
| *Paris yunnanensis* | MN686112 | MN647568 |
| *Trillium tschonoskii* | MN125577 | MN174897 |
