# Supplementary material for: Genome skimming as an efficient tool for authenticating commercial products of the pharmaceutically important Paris yunnanensis (Melanthiaceae)
Source: BMC Plant Biol. 2023 Jun 29;23:344. doi: 10.1186/s12870-023-04365-x (PMC10308783; doi:10.1186/s12870-023-04365-x)
Supplement: Supplementary file 2 — Supplementary Material 2 [file 12870_2023_4365_MOESM2_ESM.docx]

**Table S1.** Plant samples newly sequenced in this study with voucher information and GenBank accession numbers.

| Taxa | Locality | Voucher | GenBank Accessions | |
| --- | --- | --- | --- | --- |
|  |  |  | Complete plastome | Ribosomal DNA |
| *Paris liiana* | Yuanjiang, Yunnan, China | Ji et Wang004 | OP837990 | OP806312 |
| *Paris liiana* | Mile, Yunnan, China | Ji et Wang007 | OP837991 | OP806313 |
| *Paris liiana* | Mengla, Yunnan, China | Ji et Wang013 | OP837992 | OP806314 |
| *Paris liiana* | Xinping, Yunnan, China | Ji et Wang019 | OP837993 | OP806315 |
| *Paris liiana* | Maguan, Yunnan, China | Ji et Wang030 | OP837994 | OP806316 |
| *Paris liiana* | Lancang, Yunnan, China | Ji et Wang032 | OP837995 | OP806317 |
| *Paris liiana* | Eshan, Yunnan, China | ZGH01 | OP021830 | OP021493 |
| *Paris liiana* | Guangnan, Yunnan, China | ZGH03 | OP021802 | OP021494 |
| *Paris liiana* | Luoping, Yunnan, China | ZGH08 | OP021793 | OP021495 |
| *Paris liiana* | Anlong, Guizhou, China | ZGH10 | OP021804 | OP021496 |
| *Paris yunnanensis* (Typical) | Eryan, Yunnan, China | Ji YH 20211011-003 | OP837988 | OP806318 |
| *Paris yunnanensis* (syn. *Paris polyphylla* var. *nana*) | Yulong, Yunnan, China | TADCL2110-1 | OP837989 | OP806319 |
| *Paris yunnanensis* (syn. *Paris polyphylla* var. *emeiensis*) | Ludian, Yunnan, China | Ji and Xie 010 | OP837996 | OP806322 |
| *Paris yunnanensis* (syn. *Paris birmanica*) | Yingjiang, Yunnan, China | Ji, Xie and Zhou 427004 | OP021806 | OP021412 |
| *Paris yunnanensis* (syn. *Paris birmanica*) | Longchuan, Yunnan, China | Ji, Xie and Zhou 427006 | OP021797 | OP021413 |
| *Paris yunnanensis* (Typical) | Shiping, Yunnan, China | Ji, Xie and Zhou 427008 | OP021843 | OP021414 |
| *Paris yunnanensis* (syn. *Paris birmanica*) | Bhamo, Myanmar | Ji, Xie and Zhou 427009 | OP021820 | OP021415 |
| *Paris yunnanensis* (syn. *Paris daliensis*) | Weishan, Yunnan, China | Yang LF and Jin L 004 | OP021831 | OP021474 |
| *Paris yunnanensis* (syn. *Paris daliensis*) | Midu, Yunnan, China | Yang LF and Jin L 005 | OP021823 | OP021475 |
| *Paris yunnanensis* (syn. *Paris daliensis*) | Changning, Yunnan, China | Yang LF and Jin L 006 | OP021845 | OP021476 |
| *Paris yunnanensis* (syn. *Paris polyphylla* var. *emeiensis*) | Simian, Sichuan, China | Yang LF and Jin L 001 | OP021817 | OP021477 |
| *Paris yunnanensis* (syn. *Paris polyphylla* var. *emeiensis*) | Tianquan, Scihan, China | Yang LF and Jin L 002 | OP021835 | OP021478 |
