# Supplementary material for: Genome skimming as an efficient tool for authenticating commercial products of the pharmaceutically important Paris yunnanensis (Melanthiaceae)
Source: BMC Plant Biol. 2023 Jun 29;23:344. doi: 10.1186/s12870-023-04365-x (PMC10308783; doi:10.1186/s12870-023-04365-x)
Supplement: Supplementary file 3 — Supplementary Material 3 [file 12870_2023_4365_MOESM3_ESM.docx]

**Table S3.** Voucher information of commercial seedlings and processed rhizomes used in this study.

| Commercial seedlings | | Processed rhizomes | |
| --- | --- | --- | --- |
| Voucher | Locality | Voucher | Locality |
| Ji and Wang 010 | Yuanjiang, Yunnan, China | Ji et Wang 036 | Yuanjiang, Yunnan, China |
| Ji et Wang 027 | Yuanjiang, Yunnan, China | Ji et Wang 048 | Yuanjiang, Yunnan, China |
| Ji et Wang 0043 | Yuanjiang, Yunnan, China | Ji et Wang 056 | Yuanjiang, Yunnan, China |
| Ji et Wang 059 | Yuanjiang, Yunnan, China | Ji et Wang 064 | Yuanjiang, Yunnan, China |
| Ji et Wang 077 | Yuanjiang, Yunnan, China | YSY001 | Yuanjiang, Yunnan, China |
| Ji et Wang 099 | Yuanjiang, Yunnan, China | JiYH2020300 | Shimian, Sichuan, China |
| Ji et Wang 102 | Yuanjiang, Yunnan, China | JiYH2020263 | Luquan, Yunnan, China |
| Ji YH 2020297 | Yulong, Yunnan, China | Ji and Xie 003 | Lijiang, Yunnan, China |
| Ji YH 2020317 | Yulong, Yunnan, China |  |  |
| TADCL 2110-H7 | Yulong, Yunnan, China |  |  |
