# Supplementary material for: Genome skimming as an efficient tool for authenticating commercial products of the pharmaceutically important Paris yunnanensis (Melanthiaceae)
Source: BMC Plant Biol. 2023 Jun 29;23:344. doi: 10.1186/s12870-023-04365-x (PMC10308783; doi:10.1186/s12870-023-04365-x)
Supplement: Supplementary file 4 — Supplementary Material 4 [file 12870_2023_4365_MOESM4_ESM.docx]

**Table S4.** Summary of genome skimming.

| Taxon | No. of total reads | Plastome | | |  | Nuclear ribosomal (nr) DNA | | |
| --- | --- | --- | --- | --- | --- | --- | --- | --- |
|  |  | Size of plastome | No. of mapped reads | Coverage (×) |  | Size of nrDNA (bp) | No. of mapped reads | Coverage (×) |
| *Paris yunnanensis* (Ji YH 20211011-003) | 34,323,660 | 158,262 | 988,404 | 901 |  | 5,852 | 37,463 | 959 |
| *Paris yunnanensis* (Ji YH 2020297) | 34,397,296 | 158,212 | 2,174,584 | 1,979 |  | 5,852 | 48,019 | 1,230 |
| *Paris yunnanensis* (Ji YH 2020317) | 27,610,624 | 157,975 | 3,505,032 | 3,203 |  | 5,852 | 17,978 | 461 |
| *Paris yunnanensis* (TADCL2110-1) | 30,616,552 | 158,216 | 826,350 | 753 |  | 5,851 | 51,570 | 1,322 |
| *Paris yunnanensis* (TADCL2110-H7) | 32,315,962 | 158,275 | 2,165,012 | 1,975 |  | 5,852 | 27,272 | 699 |
| *Paris yunnanensis* (Ji,Xie and zhou 427004) | 26,553,746 | 158,311 | 915,857 | 873 |  | 5,852 | 23,704 | 612 |
| *Paris yunnanensis* (Ji,Xie and zhou 427006) | 32,143,170 | 158,141 | 606,956 | 579 |  | 5,852 | 29,297 | 751 |
| *Paris yunnanensis* (Ji,Xie and zhou 427008) | 29,221,908 | 158,083 | 897,669 | 857 |  | 5,852 | 25,522 | 658 |
| *Paris yunnanensis* (Ji,Xie and zhou 427009) | 29,385,010 | 158,429 | 488,802 | 466 |  | 5,852 | 30,454 | 786 |
| *Paris yunnanensis* (Yang LF and Jin L 004) | 21,030,470 | 158,142 | 631,117 | 603 |  | 5,852 | 39,111 | 1,009 |
| *Paris yunnanensis* (Yang LF and Jin L 005) | 23,412,234 | 157,950 | 712,214 | 681 |  | 5,852 | 51,801 | 1,337 |
| *Paris yunnanensis* (Yang LF and Jin L 006) | 22,251,800 | 158,112 | 520,347 | 497 |  | 5,852 | 48,756 | 1,258 |
| *Paris yunnanensis* (Yang LF and Jin L 001) | 35,730,218 | 158,118 | 1,086,216 | 1,037 |  | 5,852 | 53,358 | 1,377 |
| *Paris yunnanensis* (Yang LF and Jin L 002) | 28,811,164 | 158,194 | 813,780 | 777 |  | 5,852 | 57,626 | 1,487 |
| *Paris yunnanensis* (Ji and Xie 003) | 33,230,862 | 158,080 | 2,558,168 | 2,334 |  | 5,852 | 50,859 | 1,304 |
| *Paris yunnanensis* (Ji and Xie 010) | 29,592,590 | 158,088 | 621,062 | 566 |  | 5,852 | 52,686 | 1,351 |
| *Paris liiana* (ZGH01) | 31,699,920 | 158,004 | 6,361,657 | 6,016 |  | 5,852 | 19,202 | 492 |
| *Paris liiana* (ZGH03) | 29,083,380 | 158,063 | 3,873,541 | 3,666 |  | 5,852 | 22,158 | 568 |
| *Paris liiana* (ZGH08) | 29,813,630 | 157,714 | 3,707,869 | 3,513 |  | 5,852 | 18,104 | 464 |
| *Paris liiana* (ZGH10) | 29,982,852 | 157,712 | 5,964,155 | 5,652 |  | 5,852 | 14,860 | 381 |
| *Paris liiana* (Ji et Wang004) | 35,528,108 | 158,007 | 4,172,230 | 3,811 |  | 5,852 | 27,216 | 697 |
| *Paris liiana*  (Ji et Wang007) | 29,218,534 | 158,250 | 1,434,723 | 1,306 |  | 5,852 | 31,134 | 798 |
| *Paris liiana* (Ji et Wang010) | 29,812,242 | 158,272 | 894,516 | 815 |  | 5,852 | 20,421 | 523 |
| *Paris liiana* (Ji et Wang013) | 30,877,494 | 158,070 | 1,252,604 | 1,143 |  | 5,852 | 32,187 | 826 |
| *Paris liiana* (Ji et Wang019) | 32,713,666 | 158,384 | 959,421 | 873 |  | 5,852 | 25,198 | 646 |
| *Paris liiana*  (Ji et Wang027) | 33,545,548 | 158,036 | 1,318,425 | 1,203 |  | 5,852 | 27,688 | 710 |
| *Paris liiana*  (Ji et Wang030) | 32,366,502 | 158,187 | 1,846,160 | 1,683 |  | 5,852 | 22,974 | 589 |
| *Paris liiana* (Ji et Wang032) | 31,998,534 | 158,322 | 1,559,847 | 1,419 |  | 5,852 | 26,607 | 681 |
| *Paris liiana* (Ji et Wang036) | 28,394,018 | 158,086 | 869,780 | 795 |  | 5,852 | 34,266 | 879 |
| *Paris liiana*  (Ji et Wang043) | 31,872,506 | 158,008 | 2,179,272 | 1,990 |  | 5,852 | 18,581 | 476 |
| *Paris liiana*  (Ji et Wang059) | 30,698,952 | 158,035 | 2,697,836 | 2,466 |  | 5,852 | 28,283 | 726 |
| *Paris liiana*  (Ji et Wang077) | 30,724,142 | 158,154 | 3,507,766 | 3,202 |  | 5,852 | 17,988 | 461 |
| *Paris liiana* (Ji et Wang099) | 28,896,332 | 158,008 | 1,044,037 | 954 |  | 5,852 | 18,843 | 483 |
| *Paris liiana* (Ji et Wang102) | 33,849,274 | 158,035 | 1,847,216 | 1,687 |  | 5,852 | 38,697 | 993 |
