# Supplementary material for: Genome skimming as an efficient tool for authenticating commercial products of the pharmaceutically important Paris yunnanensis (Melanthiaceae)
Source: BMC Plant Biol. 2023 Jun 29;23:344. doi: 10.1186/s12870-023-04365-x (PMC10308783; doi:10.1186/s12870-023-04365-x)
Supplement: Supplementary file 5 — Supplementary Material 5 [file 12870_2023_4365_MOESM5_ESM.docx]

**Table S5.** Sequence characteristics of plastomes and nuclear ribosome DNA arrays generated in this study.

| Taxon | Plastome | | | |  | Ribosomal DNA | | | | | |
| --- | --- | --- | --- | --- | --- | --- | --- | --- | --- | --- | --- |
|  | Plastome size (bp) | LSC length (bp) | SSC length (bp) | IRas length (bp) |  | Ribosome size (bp) | 26S rRNA (bp) | 5.8S rRNA (bp) | 18S rRNA (bp) | ITS1 (bp) | ITS2(bp) |
| *Paris yunnanensis* (Ji YH 20211011-003) | 158,262 | 84,336 | 18,588 | 27,669 |  | 5,852 | 3,401 | 169 | 1,810 | 235 | 237 |
| *Paris yunnanensis* (Ji YH 2020301) | 157,980 | 84,228 | 18,692 | 27,530 |  | 5,836 | 3,401 | 169 | 1,810 | 220 | 236 |
| *Paris yunnanensis* (Ji YH 2020297) | 158,212 | 84,365 | 18,645 | 27,601 |  | 5,852 | 3,401 | 169 | 1,810 | 235 | 237 |
| *Paris yunnanensis* (Ji YH 2020317) | 157,975 | 84,338 | 18,645 | 27,496 |  | 5,852 | 3,401 | 169 | 1,810 | 235 | 237 |
| *Paris yunnanensis* (TADCL2110-1) | 158,216 | 84,375 | 18,557 | 27,642 |  | 5,851 | 3,401 | 168 | 1,810 | 235 | 237 |
| *Paris yunnanensis* (TADCL2110-H7) | 158,275 | 84,342 | 18,579 | 27,677 |  | 5,852 | 3,401 | 169 | 1,810 | 235 | 237 |
| *Paris yunnanensis* (Ji,Xie and zhou 427004) | 158,311 | 84,305 | 18,524 | 27,741 |  | 5,852 | 3,401 | 169 | 1,810 | 235 | 237 |
| *Paris yunnanensis* (Ji,Xie and zhou 427006) | 158,141 | 84,270 | 18,669 | 27,601 |  | 5,852 | 3,401 | 169 | 1,810 | 235 | 237 |
| *Paris yunnanensis* (Ji,Xie and zhou 427008) | 158,083 | 84,226 | 18,655 | 27,601 |  | 5,852 | 3,401 | 169 | 1,810 | 235 | 237 |
| *Paris yunnanensis* (Ji,Xie and zhou 427009) | 158,429 | 84,294 | 18,513 | 27,811 |  | 5,852 | 3,401 | 169 | 1,810 | 235 | 237 |
| *Paris yunnanensis* (Yang LF and Jin L 004) | 158,142 | 84,261 | 18,617 | 27,632 |  | 5,852 | 3,401 | 169 | 1,810 | 235 | 237 |
| *Paris yunnanensis* (Yang LF and Jin L 005) | 157,950 | 84,249 | 18,531 | 27,585 |  | 5,852 | 3,401 | 169 | 1,810 | 235 | 237 |
| *Paris yunnanensis* (Yang LF and Jin L 006) | 158,112 | 84,273 | 18,575 | 27,632 |  | 5,852 | 3,401 | 169 | 1,810 | 235 | 237 |
| *Paris yunnanensis* (Yang LF and Jin L 001) | 158,118 | 84,279 | 18,575 | 27,632 |  | 5,852 | 3,401 | 169 | 1,810 | 235 | 237 |
| *Paris yunnanensis* (Yang LF and Jin L 002) | 158,194 | 84,214 | 18,550 | 27,715 |  | 5,852 | 3,401 | 169 | 1,810 | 235 | 237 |
| *Paris yunnanensis* (Ji and Xie 003) | 158,080 | 84,306 | 18,574 | 27,600 |  | 5,852 | 3,401 | 169 | 1,810 | 235 | 237 |
| *Paris yunnanensis* (Ji and Xie 010) | 158,088 | 84,341 | 18,755 | 27,496 |  | 5,852 | 3,401 | 169 | 1,810 | 235 | 237 |
| *Paris liiana* (ZGH01) | 158,004 | 84,319 | 18,565 | 27,560 |  | 5,852 | 3,401 | 169 | 1,810 | 235 | 237 |
| *Paris liiana* (ZGH03) | 158,063 | 84,313 | 18,490 | 27,630 |  | 5,852 | 3,401 | 169 | 1,810 | 235 | 237 |
| *Paris liiana* (ZGH08) | 157,714 | 84,339 | 18,303 | 27,536 |  | 5,852 | 3,401 | 169 | 1,810 | 235 | 237 |
| *Paris liiana* (ZGH10) | 157,712 | 84,323 | 18,317 | 27,536 |  | 5,852 | 3,401 | 169 | 1,810 | 235 | 237 |
| *Paris liiana* (Ji et Wang004) | 158,007 | 84,355 | 18,532 | 27,560 |  | 5,852 | 3,401 | 169 | 1,810 | 235 | 237 |
| *Paris liiana* (Ji et Wang007) | 158,250 | 84,353 | 18,657 | 27,620 |  | 5,852 | 3,401 | 169 | 1,810 | 235 | 237 |
| *Paris liiana* (Ji et Wang010) | 158,272 | 84,435 | 18,567 | 27,635 |  | 5,852 | 3,401 | 169 | 1,810 | 235 | 237 |
| *Paris liiana* (Ji et Wang013) | 158,070 | 84,389 | 18,561 | 27,560 |  | 5,852 | 3,401 | 169 | 1,810 | 235 | 237 |
| *Paris liiana* (Ji et Wang019) | 158,384 | 84,419 | 18,585 | 27,690 |  | 5,852 | 3,401 | 169 | 1,810 | 235 | 237 |
| *Paris liiana* (Ji et Wang027) | 158,036 | 84,386 | 18,538 | 27,556 |  | 5,852 | 3,401 | 169 | 1,810 | 235 | 237 |
| *Paris liiana* (Ji et Wang030) | 158,187 | 84,316 | 18,639 | 27,616 |  | 5,852 | 3,401 | 169 | 1,810 | 235 | 237 |
| *Paris liiana* (Ji et Wang032) | 158,322 | 84,301 | 18,611 | 27,705 |  | 5,852 | 3,401 | 169 | 1,810 | 235 | 237 |
| *Paris liiana* (Ji et Wang043) | 158,008 | 84,323 | 18,565 | 27,560 |  | 5,852 | 3,401 | 169 | 1,810 | 235 | 237 |
| *Paris liiana* (Ji et Wang059) | 158,035 | 84,386 | 18,537 | 27,556 |  | 5,852 | 3,401 | 169 | 1,810 | 235 | 237 |
| *Paris liiana* (Ji et Wang077) | 158,154 | 84,329 | 18,565 | 27,630 |  | 5,852 | 3,401 | 169 | 1,810 | 235 | 237 |
| *Paris liiana* (Ji et Wang099) | 158,008 | 84,323 | 18,565 | 27,560 |  | 5,852 | 3,401 | 169 | 1,810 | 235 | 237 |
| *Paris liiana* (Ji et Wang102) | 158,035 | 84,386 | 18,537 | 27,556 |  | 5,852 | 3,401 | 169 | 1,810 | 235 | 237 |
