# Supplementary figures and images for: Genome skimming as an efficient tool for authenticating commercial products of the pharmaceutically important Paris yunnanensis (Melanthiaceae)
Source: BMC Plant Biol. 2023 Jun 29;23:344. doi: 10.1186/s12870-023-04365-x (PMC10308783; doi:10.1186/s12870-023-04365-x)

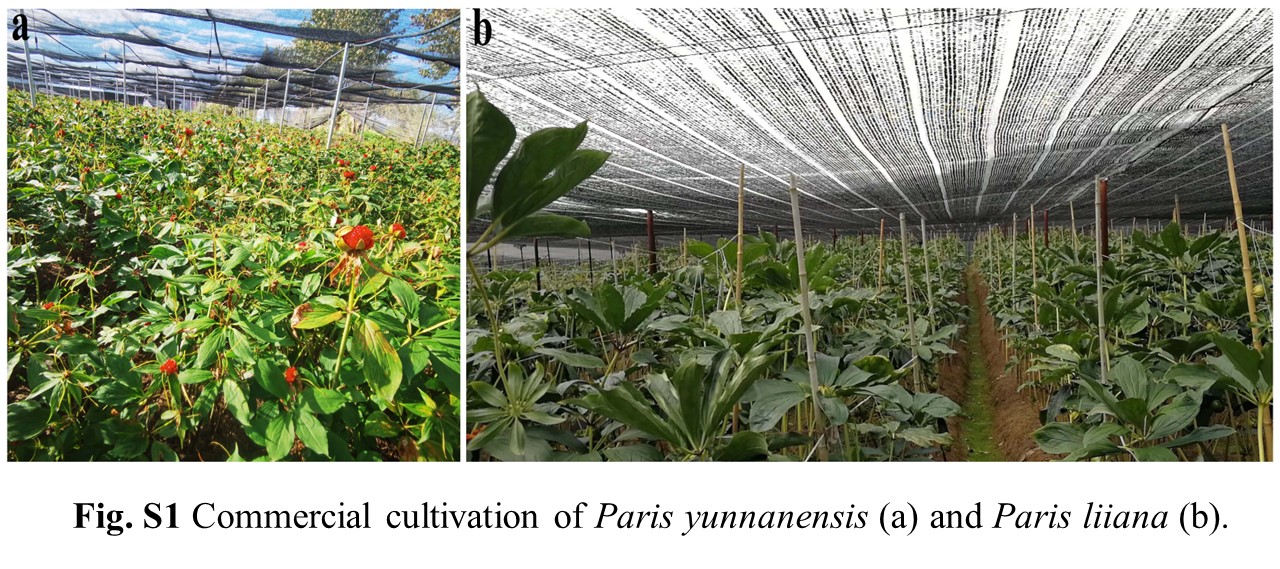

Supplement: Supplementary file 6 — Supplementary Material 6 [file 12870_2023_4365_MOESM6_ESM.jpg]
